# The development of aesthetic experience through virtual and augmented reality

**DOI:** 10.1038/s41598-024-53840-4

**Published:** 2024-02-21

**Authors:** Cong Zhou, JianQi Li

**Affiliations:** Teacher Education College, Hunan City University, Yiyang, Hunan China

**Keywords:** Aesthetic experience, Augmented reality, Higher education, Prosperity, User experience, Virtual reality, Environmental sciences, Health care

## Abstract

Emerging technological innovations offer the potential for experiential engagement through virtual scenarios, yet the viability of this approach for educational purposes remains significantly underexplored. This study aims to assess the feasibility of Augmented Reality (AR) and Virtual Reality (VR) technologies in providing users with aesthetic experiences when visiting digital exhibitions. A total of 190 students participated in this investigation. The control group visited traditional exhibits at the Palace Museum in Beijing. This group underwent a survey to evaluate their acquired aesthetic experience. In contrast, the experimental group, comprising 96 students, engaged with VR/AR scenarios at the Palace Museum in Beijing. Accordingly, students in the experimental group were also surveyed to evaluate both their aesthetic experiences and, additionally, their user experiences. The survey results unveiled significant distinctions in aesthetic experiences between students in the control and experimental groups. Moreover, there were notable correlations between individual variables related to user and aesthetic experiences within the experimental group. Furthermore, the study revealed disparities in both user and aesthetic experiences among male and female students. The findings have implications for aesthetic education teachers and officials in the context of developing sound strategies for providing aesthetic experiences to their students. This information is also of interest to employees of museums, exhibitions, and other cultural facilities, who are interested in holding or hold digital exhibitions.

## Introduction

In the twenty-first century, many educational institutions started employing digital technologies as a standard practice in teaching. Scientific publications describing interventions based on Augmented Reality (AR) and Virtual Reality (VR) have become a prominent trend in the field of education^1^. However, there is a pressing need to support the process of adapting these immersive technologies to educational needs and assess the effectiveness of their utilization by students^2^.

Numerous studies have delved into the internal and external contexts of virtual scenarios^3–5^, yet they have not touched upon the domain of aesthetic education. The focal point of this investigation lies in a hitherto unexplored terrain, specifically the acceptability of employing Virtual Reality (VR) and Augmented Reality (AR) technologies for eliciting aesthetic experiences.

Aesthetic education can lay the foundation for raising successful and prosperous individuals^6^. It focuses on a comprehensive acquaintance with art, culture, and poetic knowledge of the world by man^7^. A sense of autonomy, critical thinking, and attention to feelings contribute to the aesthetic development of an individual as a personality^7^. In the contemporary curriculum of advanced Western societies, such as the UK, the USA, Australia, New Zealand, and Canada, aesthetic education is not compulsory, while science, technology, engineering, and mathematics receive increased attention^8^. Several factors point to a definite crisis in the arts and humanities: firstly, the lack of due attention in public and political rhetoric^8^; secondly, the reduced number of subjects that are not directly related to the future profession in academic courses^8^; thirdly, the attitude to creative and aesthetic subjects as secondary at all levels of general education^9^; and fourthly, cutting funding for the arts and humanities^8^. The authors of this study suggest that to overcome the current crisis in aesthetic education, there is an urgent need to focus on the utilization of VR/AR technologies to provide users with aesthetic experiences.

### The importance of aesthetic experience in education

The origins of the concept of aesthetics date back to 1744 when the German philosopher Alexender Baumgarten first used the term “aesthetics” to refer to the science of beauty. In the “Letters on the Aesthetic Education of Man” dated 1795, the poet and playwright Friedrich Schiller postulated that the fleeting experience of beauty and the development of aesthetic taste enable a person to break the cycle of selfishness and social and material dependence^10^.

Mahgoub and Aldbesi^11^, as well as Miralay and Egitmen^12^, propose to perceive aesthetics through art. Students can learn new ways to appreciate and value art and get smart tool models to form their critical opinions^12^. There are researchers, who question certain aspects of the connection between aesthetic education and art^13^. Redfern^13^ analyzed the UK curriculum, which states that art is central to the aesthetic field, and explained her point of view by the need for empirical observations. Spivak^14^ provided a 23-year cross-section of the difficulties, joys, and paradoxes of teaching the humanities, which is nested in 25 essays. Spivak’s^14^ florid prose conveys the message that aesthetic education should be aimed primarily at expanding the imagination without replacing it with something already known^15^.

In their qualitative study, Miralay and Egitmen^12^ conducted interviews with art educators from several universities in Northern Cyprus; the questions addressed awareness, perception, and approaches to aesthetics. The main conclusions from the interviews^12^: (1) aesthetics has a philosophical and artistic meaning that changes throughout history (depending on lifestyle and social phenomena) and differs for different cultures; (2) the influence of aesthetic education on art education is undeniable and aesthetics is the raw material for art; (3) aesthetics as a systematic way of thinking and beauty that can be communicated through form, sound, color, movement, interaction, and technology; (4) aesthetic sensitivity is not only a part of art education but also of other disciplines.

Thus, previous researchers^11,12^ postulated the need to provide aesthetic education for students, and art education can be a basis for this. The levels of aesthetic perception can directly depend on the quality of faculty arts curricula^12^. Aesthetic perception can not only improve academic performance but also empower and motivate students to create works of art^12^.

Aesthetic experience is reported as a symbiosis of the conscious and subconscious that reveals the potential for artistic self-expression of the individual, improves learning outcomes, and develops creativity^16^. Researchers also reported that aesthetic experience can develop aesthetic intelligence. The latter is understood as the ability to understand, think, imagine, and create based on the gained experience^7^.

D’Olimpio^8,17^ argues that art and aesthetic education are meant to provide beautiful and touching experiences, without which life would be poor. In two recent papers, D’Olimpio^8,17^ advocates for compulsory aesthetic education within the curriculum. The author emphasizes the value of aesthetic experience provided by art as well as the role that experience plays in eudaimonia, the flourishing of life. D’Olimpio^17^ refutes the main value of aesthetic education in self-expression or moral development and the formation of students’ character. Instead, the researcher^17^ focuses on the ability of aesthetic education to offer, invite, and call for aesthetic experience.

Prosperity is defined as one of the fundamental goals of modern education^18,19^. According to Kristjánsson^19^, student life should involve engagement with self-transcending ideals and elicit enthusiasm in ways that go far beyond Aristotle's concepts of eudaimonia. Brighouse^18^ sees the foundation of a flourishing life in the ability to find joy in experience and activity. D’Olimpio^17^ does not acknowledge the fundamental role of aesthetic education in moral development. However, the author asserts that aesthetic education is necessary due to its ability to offer and evoke aesthetic experiences. During their studies, students have the opportunity to express themselves in various directions. They decide on activities that may become a mere hobby or the foundation for a successful career and a lifelong passion^20^. Engaging in activities for the sake of the activity itself, such as reading literature as an intrinsically valuable pursuit, is no less justified than an activity pursued for the sake of a grade^20^.

**Figure 1:**
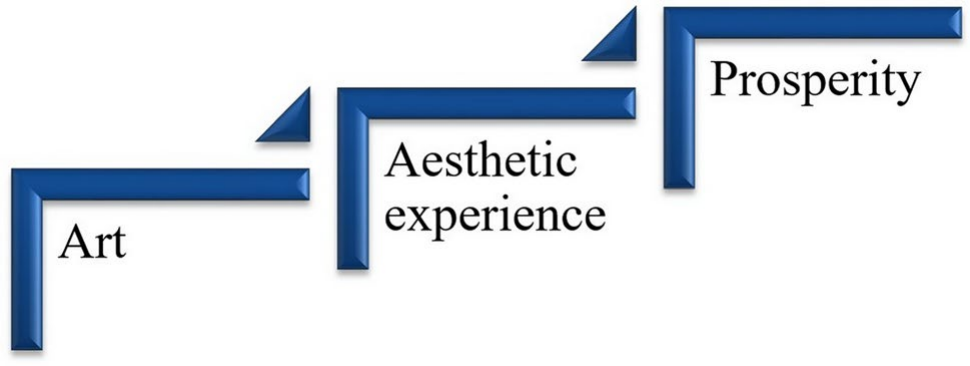
Model confirming the importance of aesthetic experience in education.

D’Olimpio^8^ sees the value of art objects in their ability to provide an aesthetic experience. This transition from the abstract concept of “Aesthetics” to the measurable concept of “Aesthetic Experience” is a turning point for those wishing to make a practical contribution to aesthetic education. The visual, sound, and aesthetic forms of art affect feelings and perception^21^. In turn, feelings and perceptions shape the aesthetic experience^22^. The aesthetic experience gained through the perception of art not only brings pleasure but also develops aesthetic taste^23^. Focus on aesthetic experience in the educational process can contribute to overall personality development, as it improves creativity^24^, critical thinking^25^, and emotional intelligence^26^. Thus, the model affirming the importance of aesthetic experience appears to be three-tiered (Fig. 1). Art (first tier) nourishes and sustains aesthetic experience (second tier), leading to flourishing as an educational goal (third tier).

The purpose of aesthetic education in the Chinese context is to cultivate the aesthetic consciousness of students, improve their aesthetic level, and stimulate their ability to create beauty^17^. Art is one of the main means of aesthetic education^17^. Every Chinese student is required to attend extensive art events on campus and learn at least 1–2 art skills in musical, visual arts, calligraphy, or local craft practices^27^. Development and immersion into various types of cultural heritage are seen as a way to personal growth and strengthening of cultural capital^28^.

### Sensation of presence, interaction, and immersion in AR/VR scenarios

Numerous prior investigations have been directed towards examining the influences of technology-related factors on the presence experience^29,30^ and usability under different visualization scenarios in virtual reality (VR)^31,32^. However, the approaches have tended to overlook the subjective nature of presence perception and interindividual variability, leaving some research gaps. Studying presence, researchers^5^ have recognized the importance of considering personality traits^5^, imaginative capabilities^4^, and emotional facets^33^.

Kober and Neuper^5^ explored the relationship between personality variables and presence in virtual reality (VR). According to the findings of Kober and Neuper^5^, impulsive tendencies, empathy, locus of control, or the Big Five personality traits exhibited heterogeneous correlations with presence, depending on the questionnaire used by the authors. Kober and Neuper^5^ determined that substantial links between personality variables and presence are impossible to comprehensively reveal through the exclusive reliance on a singular measure of presence. Hence, it is advisable to employ various metrics for assessing presence in VR and utilize an overall aggregated presence score.

Burdea and Coiffet^34^ postulated that the phenomenon of presence in a virtual environment is contingent upon three pivotal constituents: (1) immersion, signifying the capacity to detach oneself from the physical realm; (2) interaction, denoting the ability to explore and engage within the virtual milieu; and (3) imagination, representing individual cognitive abilities for mental imagery. Building upon the theoretical foundation laid forth by Burdea and Coiffet^34^, Iachini et al.^4^ undertook an examination of the nexus between perceived presence and the faculty of mental imagination. According to the findings reported by Iachini et al.^4^, the intensity of users' sensation of presence in immersive technologies was positively correlated with the vividness of their mental imagery. Furthermore, Iachini et al.^4^ reported that the ability to control mental imagery exhibited a weak correlation with the experience of presence.

Gorini et al.^3^ investigated the interrelationships among presence and technological, cognitive, and emotional factors. The findings of Gorini et al.^3^ revealed a substantial impact of narration and immersion, as well as a robust correlation between narration and immersion. The authors underscore the notion that immersion intensifies the illusion of place, while narration contributes to the generation of emotional responses and the augmentation of the subjects' sense of internal presence.

Barbot and Kaufman^35^ investigated the impact of various facets of the user experience from the perspective of empathy. Respondents in the study by Barbot and Kaufman^35^ evaluated variables of their virtual reality (VR) experience, including immersion-presence, the illusion of body ownership, the illusion of agency, and engagement. The findings of Barbot and Kaufman^35^ remained consistent across all types of experiences, with the most significant predictor of the user experience identified as the illusion of body ownership and agency^35^.

### VR/AR technologies for aesthetic experience

High-quality aesthetic education requires additional support, such as visits to creative workshops, theaters, philharmonic societies, museums, exhibitions, and galleries^36^. The students can visit them virtually without leaving the campus. Information technology has changed pedagogical approaches, making learning deeper and more interactive^37^. At present, the development of Virtual Reality (VR) and Augmented Reality (AR) allows for higher education students with an experience that connects theory and practice.

VR and AR are technological systems based on computers and devices that allow users to fully or partially immerse themselves in the world of digitized images^38,39^. VR replaces the real environment with a simulated one; it involves direct interaction between the user and the system^1^. The user is immersed in an artificial three-dimensional scenario generated by a computer and does not notice anything in the real world^38^. AR does not replace but complements reality: the user can see and interact with virtual images superimposed on a real physical environment^2^. Through updates and low-cost applications, contemporary mobile phones make AR technology an innovative space available for widespread and regular use in educational practice.

González-Zamar and Abad-Segura^1^ analyzed global research on the application of virtual reality in higher education over the past thirty years (1989–2019). The researchers reported that in recent years, especially in developed countries, VR has become an alternative to traditional learning^1^. The reason for the demand for VR is due to the three-dimensional visualization, immersion, and interaction similar to the real world^1^. When using VR/AR technologies, the cognitive activity of students occurs in a similar way to real life; it is a practical activity with a real sense of perception^17^. Through VR/AR, students get the opportunity not only to participate in the activities of aesthetic objects but also to observe the change in these objects through virtual perception^17^.

The use of multimedia equipment in the classroom does not allow for detailed and thorough multi-dimensional observation, unlike VR/AR. The VR/AR environment provides students with the opportunity to get acquainted with painting, sculpture, or architecture personally; they can create their own artwork^40^. Thus, in the research by Hui et al.^40^, schoolchildren were proposed to take art courses “Mighty General” and “Southern Song Dynasty Official Kiln” using VR. Hui et al.^40^ planned and designed the porcelain manufacturing process. The students went through all the stages of manufacturing (melting, drawing, printing, cutting, drying, glazing, and kiln firing) and printed the finished work on a 3D printer. Hui et al.^40^ noted improvements in the effects of learning in the classroom. The studied approach provided opportunities for obtaining knowledge that was unavailable before, a high concentration of attention on the learning task, and increased creativity.

Cabero-Almenara et al.^2^ assessed the learning effect of AR and VR on a sample of master’s degree students majoring in Arts. Cabero-Almenara et al.^2^ developed a 3D object shaped by the artistic expressions of the Church of the Annunciation in Seville. The intervention of Cabero-Almenara et al.^2^ aimed to determine the degree of acceptance of both AR and VR technologies and the technical and aesthetic aspects of using AR/VR. The results of Cabero-Almenara et al.^2^ indicate high acceptance of the technologies by students and the intention to reuse them.

The purpose of this research is to compare the effectiveness of using virtual and augmented reality in the context of obtaining an aesthetic experience when visiting digital exhibitions. This study aims to answer three primary research questions (RQs):

#### RQ1

Are there differences in the aesthetic experience of museum visitors who have participated in AR/VR scenarios compared to those who have not?

#### RQ2

What is the relationship between user experience and aesthetic experience?

#### RQ3

Are there variances in user experience and aesthetic experience based on demographic variables?

This article focuses on assessing the viability of AR/VR technologies in providing users with an aesthetic experience during their visits to digital exhibitions.

## Materials and methods

### Participants

All students (1st to 3rd year of studying) from the Teacher Education College of Hunan City University, who visited the Palace Museum in Beijing as part of an educational excursion, were invited to participate in the study. They received information about the experiment before the trip that was provided by their educational institution. Inclusion criteria for the study: being of legal age, visiting the Palace Museum in Beijing, providing consent to participate, and proficiency in the Chinese language. Thus, 192 individuals met the inclusion criteria; they were evenly divided into experimental and control groups. Two participants from the control group declined to complete the questionnaires. Consequently, 96 students from the experimental group and 94 students from the control group filled out the surveys. Their demographic information is presented in Table 1.

**Table 1:** Demographic data of participants.

	Experimental group (n = 96)		Control group (n = 94)	
	n	%	n	%
Sex				
Male	42	44	45	48
Female	54	56	49	52
Age		0		0
18–20	33	34	35	37
21–23	38	40	37	39
24–26	25	26	22	23

### Procedure

The students of the Teacher Education College of Hunan City University visited the Palace Museum in Beijing. It is the world’s largest palace complex with over 1.86 million relics. Since 2000, the Palace Museum has used virtual technology. During this time, it was possible to collect a large database of digital models of architecture and art (sculptures, ceramics, bronze, glass, gold and silver, and jade) and present them in the museum. Through interactive displays, museum visitors can get comprehensive information about the creation, design, and use of the works of art. Students from both groups visited the Hall of Mental Cultivation and explored the traditional museum exhibitions. The traditional visit took 1.5 h. Immediately after concluding the traditional visit, students from the control group completed the questionnaires. Their participation in the study concluded at that point.

**Figure 2:**
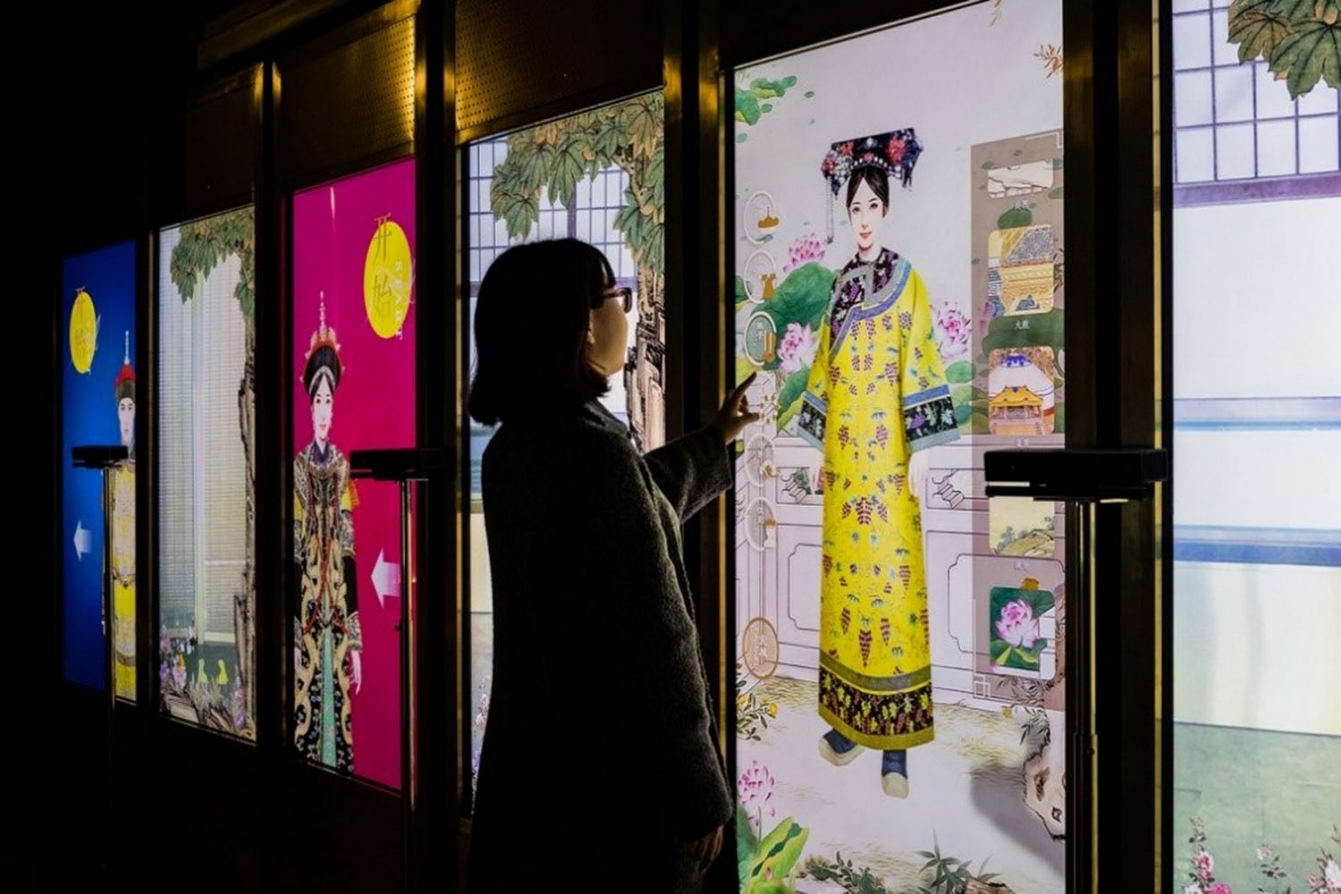
Imperial attire. *Source* The Palace Museum in Beijing.

Subsequently, the students in the experimental group visited the AR Imperial Attire exhibition and explored the Hall of Mental Cultivation using VR. Museum staff provided technical support for the experience, explaining the details and addressing all questions. The AR experience allowed them to try on Qing dynasty costumes (Rong Suit, Ji Suit, Dress Suit, and Casual Suit). Qing dynasty clothing combines traditional Manchu elements with traditional Han designs. For the AR experience, body gesture recognition technology and a 3D body-sense camera were the tools that enabled virtual try-ons. The screen content could be interactively controlled. Users not only receive information about the clothes of the Qing dynasty but also control the fitting and wave their arms and legs in palace costumes against various backgrounds of the Palace Museum (Fig. 2).

**Figure 3:**
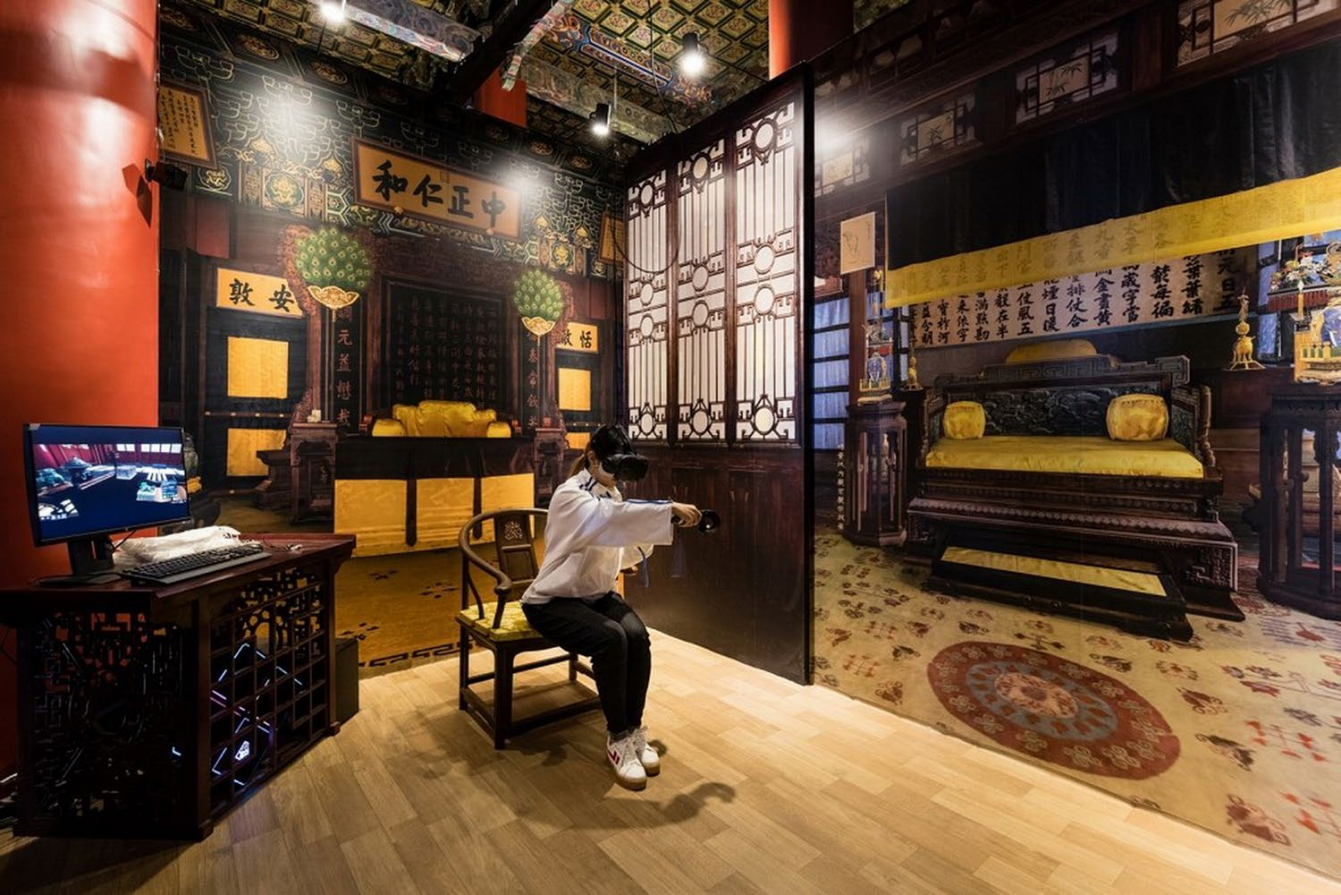
The hall of mental cultivation. *Source* The Palace Museum in Beijing.

For the VR experience, the tool was VIVE Pro HMD, featuring AMOLED (Dual AMOLED 3.5 diagonal screens with a 110° field of view, and a resolution of 2880 by 1600 pixels. The setup also included SteamVR base stations and headphones. By wearing the headset and sitting in a moving seat, the participants followed the Ming Dynasty Emperor, Zhu Di, through the palace and listened to narratives about the palace's architecture, construction, and rituals (Fig. 3). The high-resolution screens and user-friendly headset ensured comfort during immersion in the cultural treasures of the Hall of Mental Cultivation. The Hall of Mental Cultivation was built in 1536 during the Ming dynasty. The students had the opportunity not only to study the cultural treasures and traditional architecture in detail and visit the place, where the emperors lived and worked but also to decorate the hall according to their tastes.

Each scenario was allocated up to 10 min for each participant, although participants had the option to discontinue their participation at any moment. A brief introductory instruction before each experience took approximately 5 min. After all participants in the experimental group had completed their experiences, the study authors invited them to complete the questionnaires. The questionnaire session lasted approximately 25 min.

All methods followed the relevant guidelines and regulations of the Declaration of Helsinki. The ethics committee of Hunan City University approved the research procedure (protocol TB 45,788,975). All participants signed informed consent.

### Scales

This study utilized two scales: The Aesthetic Experience Scale (AES)^41^ and the Presence Questionnaire (PQ)^42^. AES is based on the dynamic relationship between the person and the object of aesthetic experience based on philosophical and psychological ideas. In her study, Stamatopoulou^41^ searched for measurable components and essential features of aesthetic experience. Awareness of the aesthetic experience of Stamatopoulou^41^ goes beyond beauty and works of art. In her model of aesthetic experience, Stamatopoulou^41^ relies on sympathetic identification, affective responses experienced contemplatively, and the concept of arousal linking emotional and cognitive processes. The model of Stamatopoulou^41^ is philosophical and psychological. Its main starting points are the following:

- Expressive perception is closely related to positive or negative forms of sympathy.
- Sensory and perceptual signals and cognitive elements interact to form a synthesized whole.
- Perceptual and conceptual structures are not identical; the latter can be better understood by rethinking, which determines the actual attitude to the stimulus.

Emotional closeness, emotional distance, and the final emotional state of relief (catharsis) are also important; emotions are the result of any perceptual and cognitive processes within the aesthetic experience.

To activate the aesthetic experience, a special state of mind is needed, which has passed the stages of activity orientation, increased suspension, and high arousal.

The AES scale^41^ consists of 28 items grouped into five categories: Cognitive Synergies and Elaboration, Emotional Closeness, Experiential Emotional Distancing, Paratelic Mode, and Expressive Perception (Table 2). Cognitive Synergies and Elaboration implies cognitive processing resulting from the impact of the object of attention^41^. Emotional Closeness characterizes the emotional sense resulting from previous emotional experiences^41^. Experiential Emotional Distancing is characterized by awareness of the aesthetic experience, followed by evaluative cognitions^41^. Paratelic Mode focuses on activity and qualitatively measures the aesthetic experience as a meta-motivational mode^41^. Expressive Perception indicates harmony between perception and emotions/motives/memories^41^. For answers, a 5-point Likert-type scale was used: (1) Never, (2) Rarely, (3) Sometimes, (4) Often, and (5) Very Often.

**Table 2:** AES.

AES Category	Questions	Cronbach’s Alpha
Evaluate how much your prior knowledge has improved your perception of the exhibits:		
Cognitive Synergies and Elaboration	Painting	0.64
	Weaving	
	Calligraphy	
	Ceramics and bronze	
	Manuscripts and historical documents	
	Jewelry	
Evaluate your agreement with the following statements:		
Emotional Closeness	I felt satisfied being surrounded by the exhibits of art in the museum	0.63
	The exhibits corresponded to my interests	
	I felt an emotional lift when I saw some of the exhibits	
	I showed emotions, including tears, while watching works of art, which especially touched me	
	I found that my body was moving to the beat of the music playing in the halls of the museum	
	I was not paying attention to the time when participating in aesthetic activities	
Experiential Emotional Distancing	The architecture of the Chinese emperors became more understandable	0.74
	Precious stones and jewelry from imperial collections became more understandable	
	Books and films about the life of Chinese emperors became more understandable	
	The paintings and sculptures echoed my expectations or previous knowledge	
	Ceramics and bronze echoed my expectations or previous knowledge	
Paratelic Mode	I have a desire to tell my friends about my experience	0.74
	I have a desire to show my friends what I have seen	
	I realize that the works of art influenced my mood and perception	
	I feel a desire to create an artwork reflecting what I have seen	
Expressive Perception	I felt free to express my reactions to the exhibits	0.63
	The works of art in the museum helped me express my own emotions and feelings	
	I enjoyed trying to identify the feelings depicted on the faces in the portraits in the museum collection	
	I felt completely immersed in the artworks or music presented in the museum	
	The visit to the museum inspired my creativity	
	I have rethought the values of documents and materials related to the history of China and the life of the emperors	
	The emotions and reactions experienced during the visit did not contradict my expectations	

The PQ^42^ assessed the user experience in the virtual environment. The PQ items can be grouped into four dimensions: representing involvement (9 items), interface quality (2 items), adaptation/ immersion (6 items), and visual fidelity (2 items). Each dimension showed adequate reliability in this study (α = 0.83, α = 0.74, α = 0.70, and α = 0.79). Each item on the PQ was assessed on a 7-point Likert scale from 1 (not at all) to 7 (completely).

### Data analysis

The tests of data for normality of distribution using the histogram showed that the data were normally distributed for parametric tests^43^. To discern differences in aesthetic experience between visitors who participated (experimental group) and those who did not (control group) in AR/VR scenarios, the study used an independent Student’s t-test. Effect sizes (Cohen's d) were calculated using the online calculator Psychometrica. Effect size interpretation followed the guidelines of Sullivan and Feinn^44^: Small Effect = 0.2, Medium Effect = 0.5, Large Effect = 0.8.

The relationship between user experience and aesthetic experience was assessed using the Pearson correlation coefficient. For each variable of user and aesthetic experience, the mean was calculated between the AR and VR experiences. Gender and age differences in user and aesthetic experiences were evaluated using a one-way analysis of variance (ANOVA). Effect sizes for ANOVA were determined by calculating partial eta squared. Effect size interpretation followed Richardson's^45^, guidelines, where small, medium, and large effects would be reflected in values of partial eta squared of 0.0099, 0.0588, and 0.1379, respectively. Data analysis involved SPSS version 23.0 statistical software.

### Ethics approval and informed consent

The research procedure was approved by the ethics committee of Hunan City University (protocol TB 45,788,975). Informed consent was signed by participants.

## Results

### RQ1: Are there differences in the aesthetic experience of museum visitors who have participated in AR/VR scenarios compared to those who have not?

The independent samples Student's t-test determined differences in aesthetic experience between visitors participating in AR/VR scenarios and the control group, i.e., students who visited the same traditional exhibitions but did not engage in AR/VR scenarios. The experimental group statistically significantly outperformed the control group across four AES constructs: Cognitive Synergies and Elaboration (t = 4.38, *p* = 0.000), Emotional Closeness (t = 3.11, *p* = 0.01), Experiential Emotional Distancing (t = 3.25, *p* = 0.001) and Paratelic Mode (t = 2.96, *p* = 0.043) (Table 3). The effect size was strong for Cognitive Synergies and Elaboration (d = 0.817), close to medium for Emotional Closeness (d = 0.525) and Experiential Emotional Distancing (d = 0.650), but relatively weak for Paratelic Mode (d = 0.295) (Table 3).

**Table 3:** Descriptive statistics and t-statistics of AES results for experimental and control groups.

Category AES	Experimental		Control		t	*p*	d
	Mean	SD	Mean	SD			
Cognitive Synergies and Elaboration	3.75	0.45	3.34	0.55	4.38	0.000*	0.817
Emotional Closeness	3.93	0.57	3.62	0.61	3.11	0.01*	0.525
Experiential Emotional Distancing	4.21	0.61	3.81	0.62	3.25	0.001*	0.650
Paratelic Mode	4.12	0.49	3.96	0.59	2.92	0.043*	0.295
Expressive Perception	3.27	0.63	3.26	0.74	0.96	0.16	0.015

*Significant at *p* < 0.05.

### RQ2: What is the relationship between user experience and aesthetic experience?

The correlation analysis was conducted on data from the experimental group students, as the control group did not undergo the PQ survey (as they did not partake in the AR/VR experience). Table 4 presents the results of the correlation analysis. There is a significant positive correlation between the following: Emotional Closeness and Representing Involvement (r(96) = 0.297, *p* = 0.019), Emotional Closeness and Adaptation/ Immersion (r(96) = 0.266, *p* = 0.022). In other words, the higher the degree of involvement in the AR/VR scenario (Representing Involvement) and the degree of adaptation to the virtual environment (Adaptation/ Immersion), the greater the participants’ emotional closeness and enjoyment of the aesthetic activity. Furthermore, a significant positive correlation was identified between Cognitive Synergies and Elaboration and Interface Quality (r(96) = 0.239, *p* = 0.026), as well as between Cognitive Synergies and Elaboration and Visual Fidelity (r(96) = 0.251, *p* = 0.014). Accordingly, the higher the quality of the interface and its visual accuracy, the greater the cognitive processing of formal and semantic structures. Additionally, Interface Quality also significantly positively correlates with Experiential Emotional Distancing (r(96) = 0.245, *p* = 0.005). This result suggests that the quality of the interface affects how easily observers can distance themselves and make objective assessments. Furthermore, a significant correlation was found between Adaptation/ Immersion and Paratelic Mode (r(96) = 0.227, *p* = 0.031) and between Adaptation/ Immersion and Expressive Perception (r(96) = 0.198, *p* = 0.037). This fact implies that the higher a user's adaptation to the features of the virtual environment, the greater the intensity of their pleasant aesthetic experience (Paratelic Mode) and the assimilation of dynamic properties of perceived objects (Expressive Perception).

**Table 4:** Descriptive statistics and correlation between PQ and AES scales.

	Mean	SD	Representing involvement	Interface quality	Adaptation/immersion	Visual fidelity
Cognitive Synergies and Elaboration	3.64	0.73	0.077	0.239*	− 0.206	0.251*
Emotional Closeness	3.99	0.74	0.297*	0.035	0.266*	0.084
Experiential Emotional Distancing	4.24	0.75	-0.021	0.245*	0.123	0.104
Paratelic Mode	4.09	0.74	0.137	0.125	0.227*	0.119
Expressive Perception	3.31	0.73	0.206*	0.102	0.198*	0.085
Mean			4.49	4.98	5.13	5.41
SD			0.56	0.64	0.71	0.60

*Significant at *p* < 0.05.

Finally, there was a significant negative relationship between Adaptation/ Immersion and Cognitive Synergies and Elaboration (r(96) = − 0.206, *p* = 0.017). Thus, cognitive processing weakens as the user adapts to the AR/VR system. No statistically significant relationships were observed among the other constructs.

### RQ3: Are there differences in user and aesthetic experiences based on respondents' demographic characteristics?

The one-way ANOVA revealed no significant differences between the responses of participants from different age groups. However, both user experience and aesthetic experience significantly differ between men and women.

The effect was significant only for the variable 'sex.' For PQ F (1, 94) = 10.41, *p* = 0.0001, with a medium effect size (partial eta squared = 0.061) (Table 5); for AES F (1, 94) = 8.26, *p* = 0.02, but the effect size was small (partial eta squared = 0.012) (Table 5).

**Table 5:** One-way ANOVA for participant demographics.

Variable	M ± SD	F	*p*	Partial eta squared	M ± SD	F	*p*	Partial eta squared
PQ					AES			
Age		2.11	0.18	0.009		1.78	0.34	0.0013
18–20	5.06 ± 0.66				3.91 ± 0.56			
21–23	4.89 ± 0.63				3.88 ± 0.51			
24–26	5.12 ± 0.51				3.87 ± 0.55			
Sex		10.41	0.0001*	0.061		8.26	0.02	0.012
Male	5.13 ± 0.59				3.63 ± 0.60			
Female	4.76 ± 0.52				4.11 ± 0.49			

## Discussion

According to the research findings, AR and VR technologies may represent a fairly strong aesthetic experience. Differences in the aesthetic experience of students participating and not participating in AR/VR scenarios (RQ1) are significant. There are significant positive correlations between Emotional Closeness and Representing Involvement, Emotional Closeness and Adaptation/ Immersion, Cognitive Synergies and Elaboration and Interface Quality, Cognitive Synergies and Elaboration and Visual Fidelity, Interface Quality and Experiential Emotional Distancing. Significant positive correlations are also observed between Adaptation/ Immersion and Paratelic Mode, Adaptation/ Immersion, and Expressive Perception. At the same time, the study showed a significant negative correlation between Adaptation/ Immersion and Cognitive Synergies and Elaboration (RQ2). The aesthetic experience of men differs from the aesthetic experience of women (RQ3).

Some studies report positive results from the use of VR/AR in educational contexts. The benefits include improved academic outcomes, personal motivation, interest in the subject, and involvement in the learning process^46,47^. The issue of improving learning outcomes is one of the most studied by researchers^6,48–50^. Some of them focus on cognitive skills (memorization and understanding) related to visualization, illustration of abstract concepts, and VR/AR multimodality^48,51^. Some others report improved motor coordination and other physical skills that allow for learning a craft or driving^50^. The current research contributed by reporting the potential of VR/AR to provide strong aesthetic experiences in the context of aesthetic education. The research showed differences between the VR/AR group and the control group in four of the five AES categories. The differences concerned Cognitive Synergies and Elaboration, Emotional Closeness, Experiential Emotional Distancing, and Paratelic Mode (Table 3). Differences between the groups by Cognitive Synergies and Elaboration suggest that the VR/AR group gained a more in-depth understanding of cultural and art subjects. In turn, students in the control group possibly experienced more superficial influence and limited interaction with the object of attention. Earlier studies have reported that AR and VR technologies may offer broader and more interactive opportunities for interacting with objects^52^. Therefore, cognitive processing works better with VR/AR. Heid^53^ argued that aesthetic experience results from a deep impact of sensory perception, which increases cognitive abilities. Higher Emotional Closeness in the VR/AR group indicates that they had stronger emotional reactions and emotional closeness to the art objects they studied. Shin^54^ has previously described the ability of AR and VR technologies to provoke empathy and greater involvement in the context of a studied object.

Superior Experiential Emotional Distancing in the group using VR/AR can be due to more conscious attention to the aesthetic experience. Participation in immersive virtual scenarios can shorten the distance between the visitor and the exhibit^55^; it could reduce experimental emotional alienation, making the experience more enjoyable. Paratelic Mode reflects the quality of the experience and the desire to share the quality experience. The VR/AR experience had richer sensory and kinesthetic stimulation, contributing to a more intense and dynamic aesthetic experience and the desire to share it.

Researchers associate motivation increase with VR/AR interactivity, which allows for autonomy and independent learning^56^, as well as gamification techniques^57^. Parong and Mayer^58^ studied the impact of VR on the motivation, engagement, interest, and performance of college students. The experiment by Parong and Mayer^58^ involved students studying the construction of the human body in immersive virtual reality or using a self-contained PowerPoint slideshow on a desktop computer. The results showed that the students, who watched the slideshow, showed significantly better results on the post-test than the students in the VR group. However, their ratings of motivation, interest, and engagement were lower^58^. Thus, increased motivation and engagement with virtual technologies are not yet a guarantee of successful learning^58^. In the current research, the high scores in the Paratelic Mode category for both technologies (AES scale) indicate the motivation of the participants to have an aesthetic experience. The authors agreed to measure the aesthetic experience and not the performance in a particular academic discipline. Therefore, in this case, there is a disparity between the conclusions of Parong and Mayer^58^, and the use of virtual technologies in aesthetic education was effective in providing an aesthetic experience.

Other benefits of VR/AR include fostering soft skills, safety, and health protection, saving time and costs, as well as adapting to individual and special needs^59^. It is reported that skills such as problem-solving, decision-making, teamwork, management, and leadership can be trained in the VR/AR environment^59^. Abdullah et al.^46^ used VR to visualize and simulate biodiversity narratives and realistic scenarios in 3D. According to Abdullah et al.^46^, virtual worlds can improve group work and independent learning skills.

Many researchers of contemporary technologies report such benefits as saving time and costs as well as adapting to individual and special needs^59^. However, in the context of VR/AR, students can significantly reduce the necessary practice in real conditions by replacing it with a virtual one (for example, preparing, restarting, and observing machines and mechanisms). The reported shortcomings of VR/AR include technical problems related to brightness, response time, resolution, and power consumption. Too low brightness reduces the display quality. In a high-brightness environment, outside light affects the low-brightness screen^60^. In addition, when using VR, the delay while changing the viewing angle makes the user dizzy^60^. Pixels on the screen at low resolution also cause dizziness and reduce the experience quality^60^. Technical issues, glitches, poor fidelity, and inconvenient headsets weaken interaction, disrupt presence, and reduce motivation to use this technology^59^. In some studies, participants mention the limitations of haptic feedback as one of the main reasons for favor of other teaching methods over VR^50^. The VR device is mounted on the head and can cause fatigue if it is too heavy; in addition, it often overheats during operation so it cannot be used continuously for a long time^60^. In the current research, the authors did not categorize technical difficulties and the respondents reported significantly better technical performance and ease of using AR compared to VR (Table 4). The Palace Museum uses high-end simulations and a high-immersion virtual reality system. Therefore, there is a need to mention not the technical problems of the devices but rather the technical difficulties of using a particular technology.

At the same time, AR users face the need to hold the smartphone, thus one hand is occupied and tactile interaction is limited^49^. Obviously, in the current research, the respondents still found it more convenient to cope with tasks using AR. However, performing more complex manipulations, as in the paper by Sanfilippo et al.^49^, can be problematic in an AR environment.

Museums using AR and VR can make a significant contribution to all components of the model confirming the importance of aesthetic experience in education (Fig. 1): art, aesthetic experience, and prosperity. The contribution to art is primarily access to a large variety of collections. The use of immersive technologies expands access to art by providing the opportunity to visit virtual exhibitions and collections from various museums around the world. Therefore, visitors can see a wide range of artistic works that can include different styles, eras, and cultures. The contribution to aesthetic experience lies in immersive perception. Visitors can find themselves inside the object of study and even recreate the environment in which the object was created. This feature enhances aesthetic perception and creates a deeper connection with the work of art. As for prosperity, the development of creativity (virtual experience can become a source of inspiration and stimulate creative thinking) and intercultural exchange.

### Research limitations

This study has certain limitations. Firstly, it focused on a single example of using AR/VR technologies to provide users with an aesthetic experience within the context of museum visits. Devices with different technical specifications and content might yield different results. The researchers acknowledge that discomfort from VR headsets or poor visualization could significantly impair the aesthetic experience. Additionally, the sample consisted exclusively of Chinese students, and the experiment is based on traditional Chinese artifacts and works of art, lacking broad geographic coverage. Further research is needed to explore the effectiveness of immersive technologies in providing aesthetic experiences across samples of different nationalities.

Another limitation of this study is its focus on short-term effects. It fails to provide insight into the long-term nature of the aesthetic experience. Future researchers may devote efforts to exploring temporal variations in the aesthetic experience facilitated by immersive technologies during museum visits for educational purposes.

## Conclusion

This study advocates for the inclusion of art as a means of developing aesthetic experience in education for subsequent success and flourishing. The study found differences between the VR/AR group and the control group in four of the five AES categories. This result suggests that the use of VR/AR effectively stimulates cognitive processes and deepens the understanding of studied subjects (Cognitive Synergies and Elaboration), improving emotional perception and a sense of closeness to art (Emotional Closeness). In addition, it reduces emotional alienation and makes aesthetic experience more positive (Experiential Emotional Distancing). As a result, these technologies allow for a more complete perception, a more interesting experience, and higher engagement (Paratelic Mode).

The paper discusses the technological aspects of using AR/VR and their connection with aesthetic experience. The revealed positive correlations indicate that stronger emotional closeness increases the sense of participation. In turn, a deeper cognitive experience implies a more satisfactory interaction with the interface and the reliability of visual elements. The negative correlation between Adaptation/ Immersion and Cognitive Synergies and Elaboration suggests that deeper immersion may reduce the cognitive activity of the user. These findings can have practical importance for designers and software developers in the development of museum exhibitions and entertainment for educational purposes. The study found that the aesthetic experience differs depending on gender. This conclusion provides additional data on the nature of aesthetic experience in virtual scenarios. Therefore, the paper contributes to understanding the impact of technology on human perception and interaction with art. Further research can combine aesthetic education and cultural heritage in the context of obtaining aesthetic experience. It is also necessary to focus on issues of self-expression and moral formation of the individual, which this study did not explore.

### Implications for practitioners

The study's findings yield two crucial insights that can be applied by designers in creating aesthetic experiences during visits to cultural heritage sites. Firstly, the greater the degree of engagement in the AR/VR scenario and adaptation to the peculiarities of the virtual environment, the higher the participants' emotional closeness and enjoyment of the aesthetic activity. The second observation, which is no less significant, is that high interface quality and visual accuracy facilitate cognitive processing. As a result, it is easier for participants to distance themselves and provide objective evaluations.

## Data Availability

The datasets used and(or) analyzed during the current study are available from the corresponding author upon reasonable request.
